# Supplementary figures and images for: Systematic identification of cell size regulators in budding yeast
Source: Mol Syst Biol. 2014 Nov 19;10(11):761. doi: 10.15252/msb.20145345 (PMC4299602; doi:10.15252/msb.20145345)

A

## Extended G1

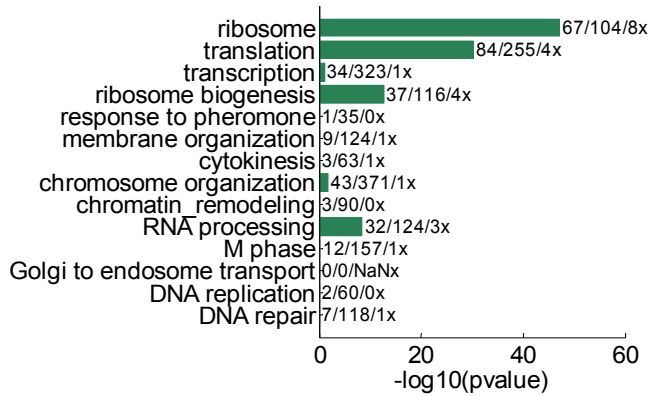

B

## Extended G2

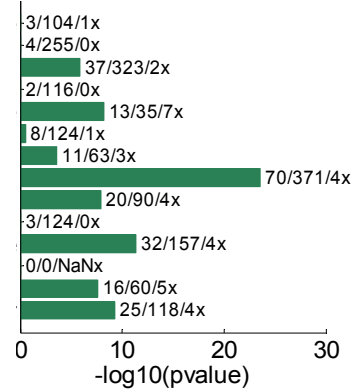

C

## Small

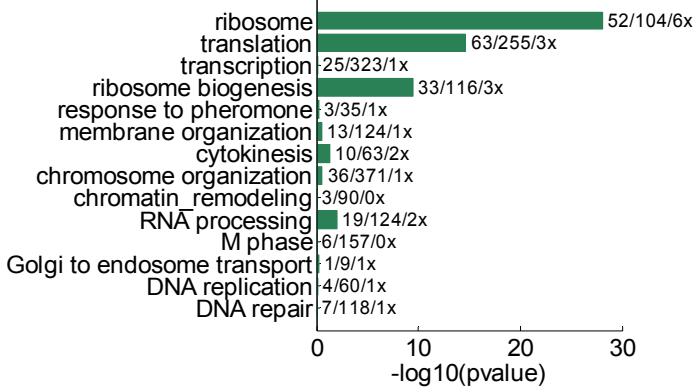

D

## Large

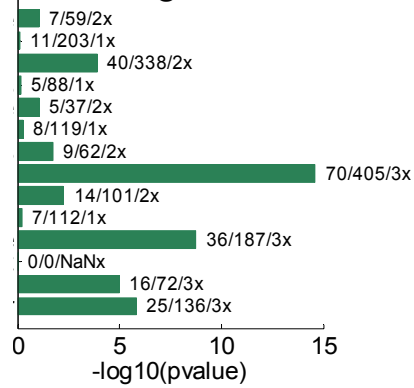

E

## Complex cell cycle

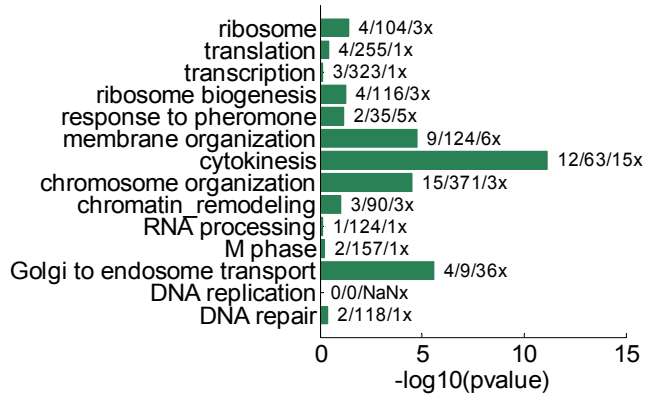

F

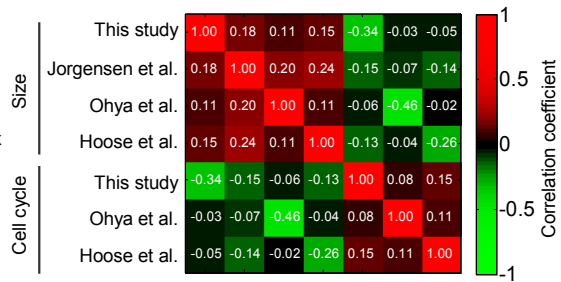

Supplement: Supplementary file 1 — Supplementary Figure S1 [file msb0010-0761-sd1.pdf]

A

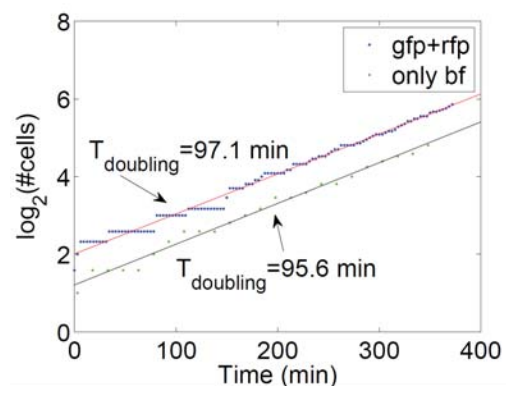

B

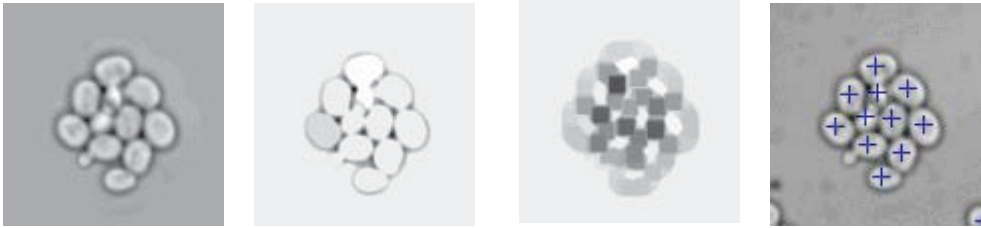

Supplement: Supplementary file 2 — Supplementary Figure S2 [file msb0010-0761-sd2.pdf]

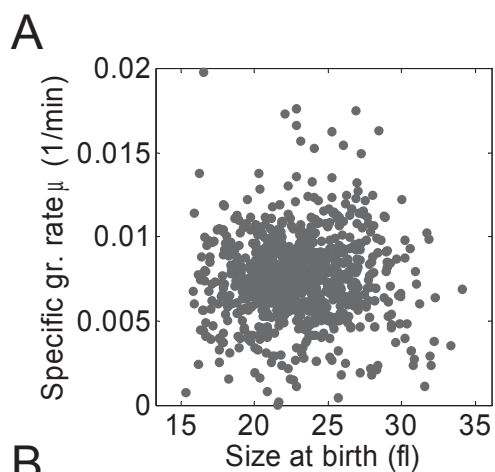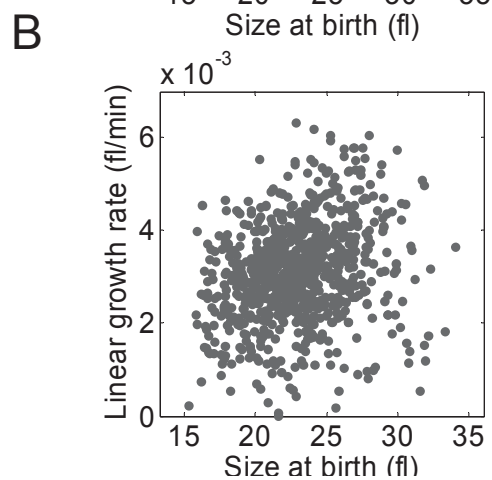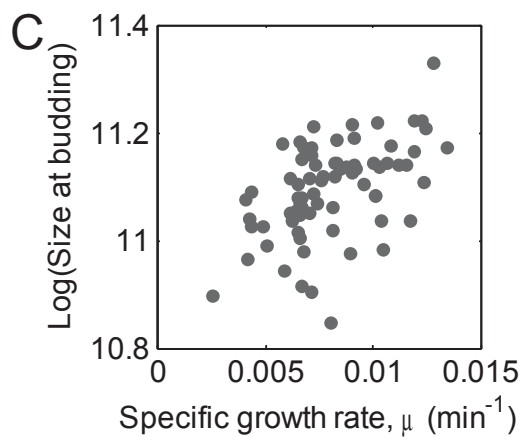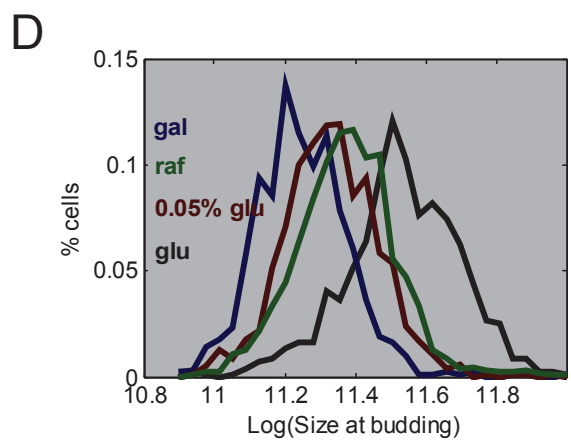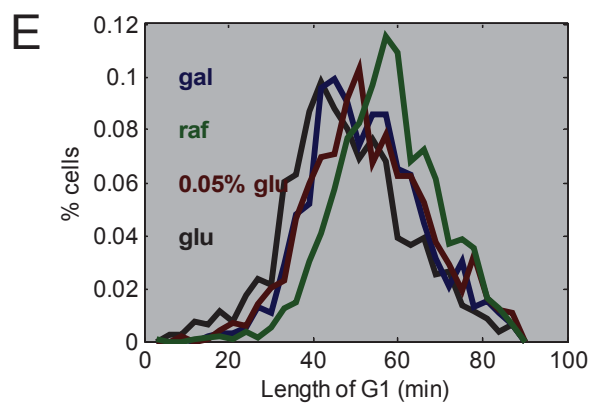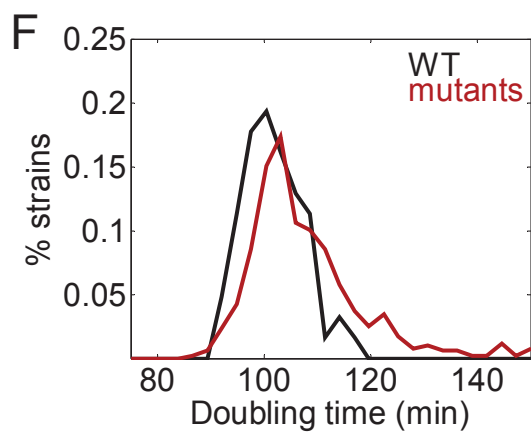

Supplement: Supplementary file 3 — Supplementary Figure S3 [file msb0010-0761-sd3.pdf]

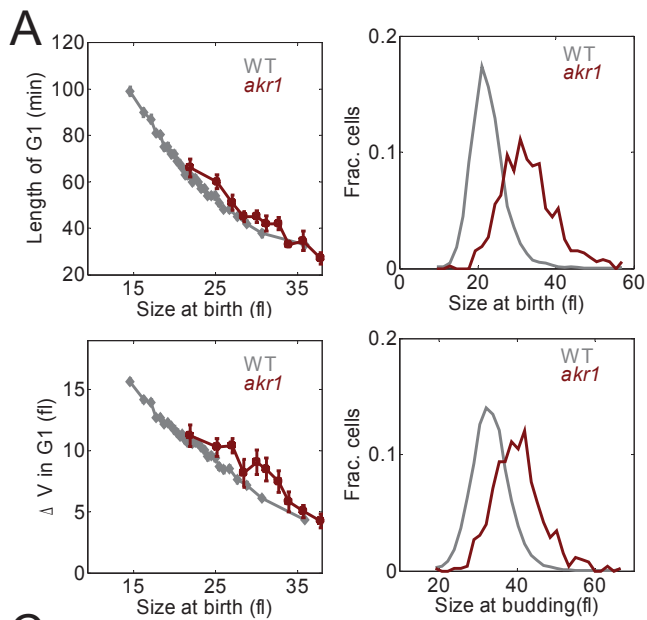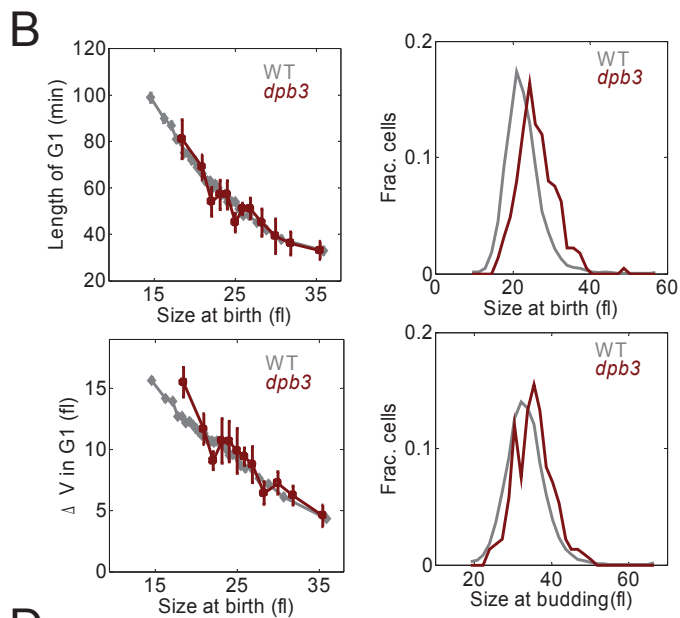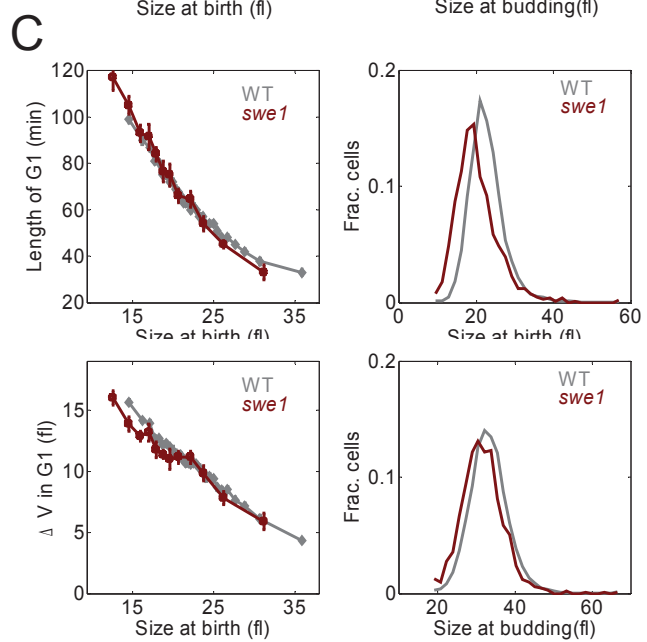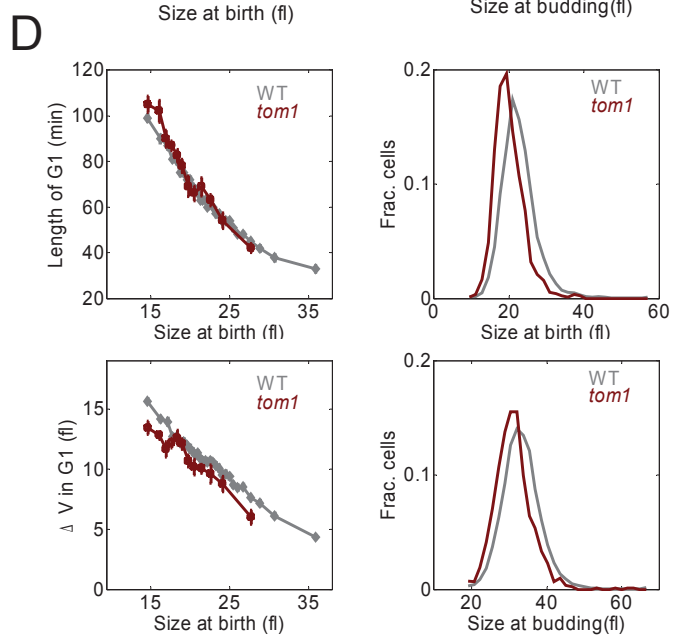

Supplement: Supplementary file 5 — Supplementary Figure S5 [file msb0010-0761-sd5.pdf]

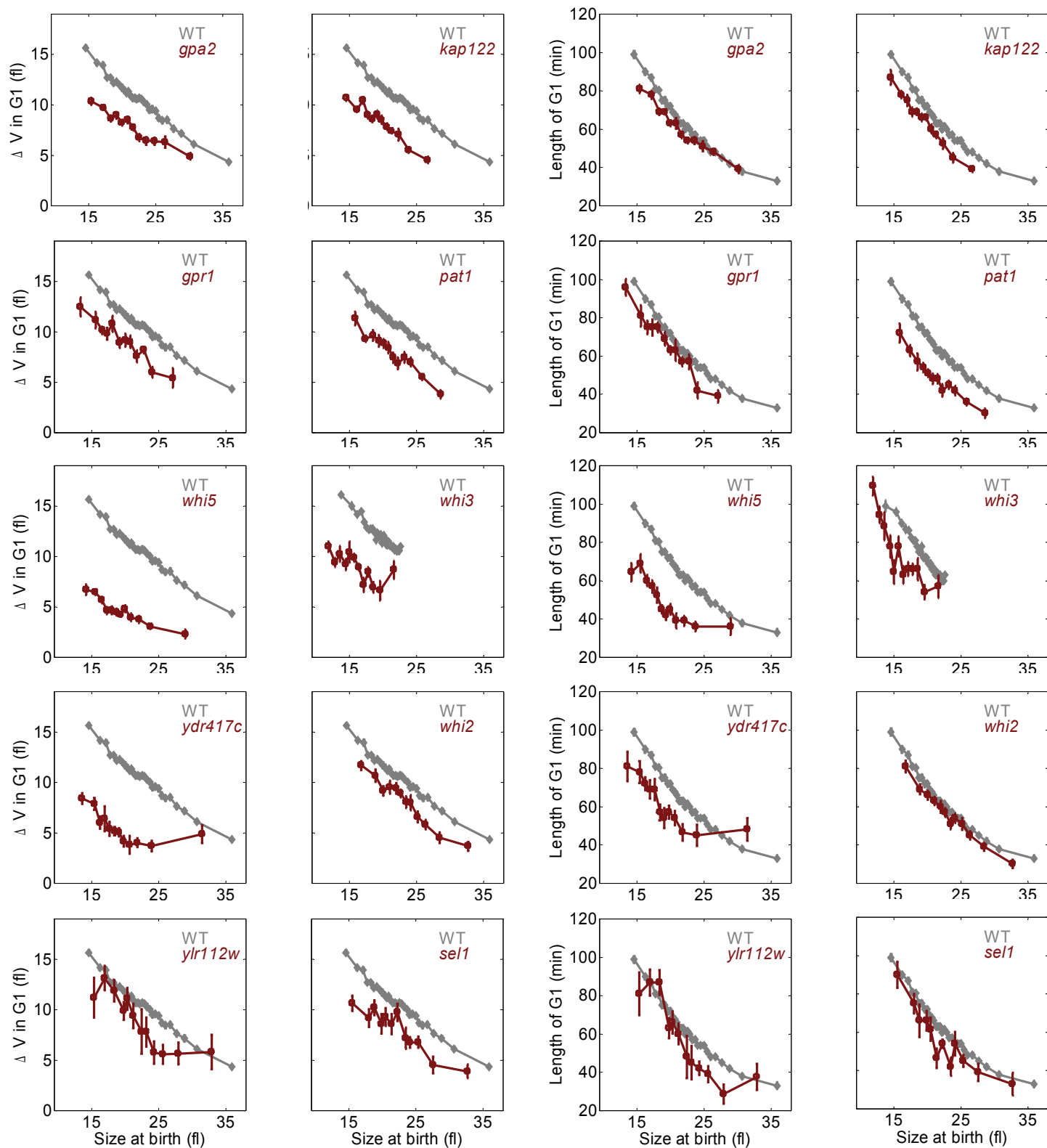

Supplement: Supplementary file 6 — Supplementary Figure S6 [file msb0010-0761-sd6.pdf]

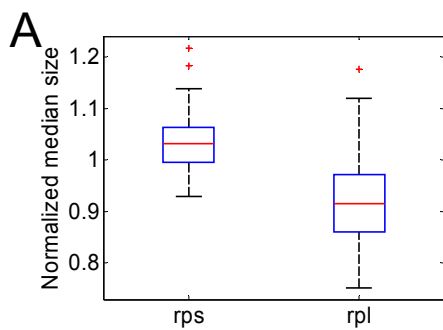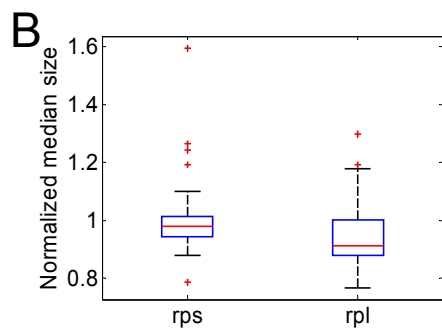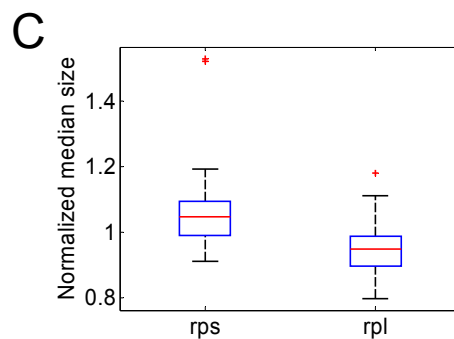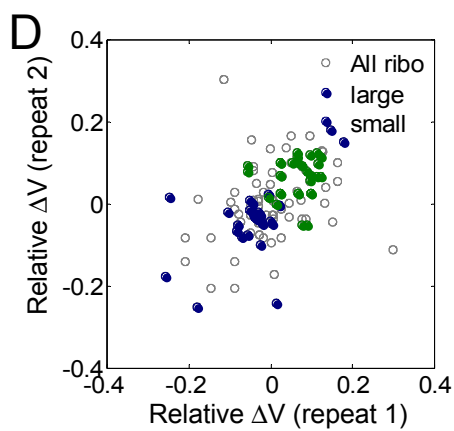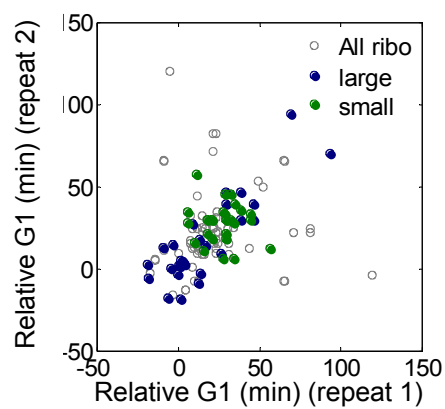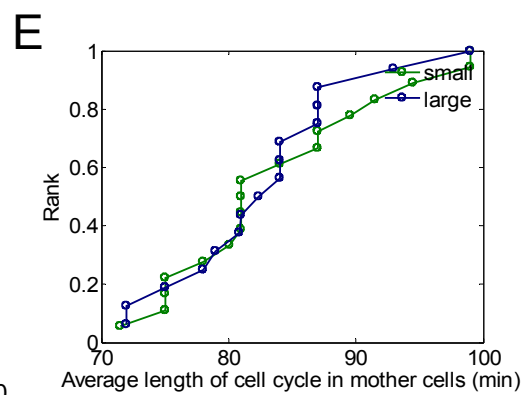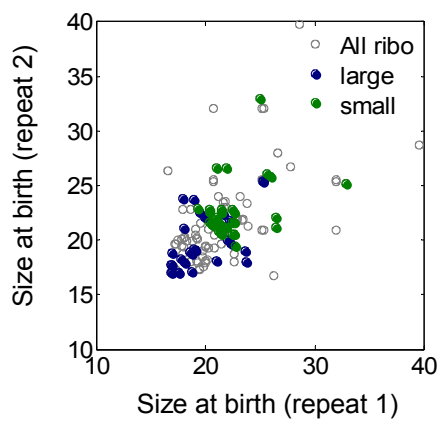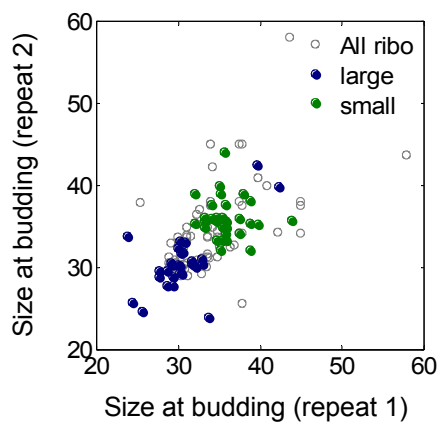

Supplement: Supplementary file 7 — Supplementary Figure S7 [file msb0010-0761-sd7.pdf]

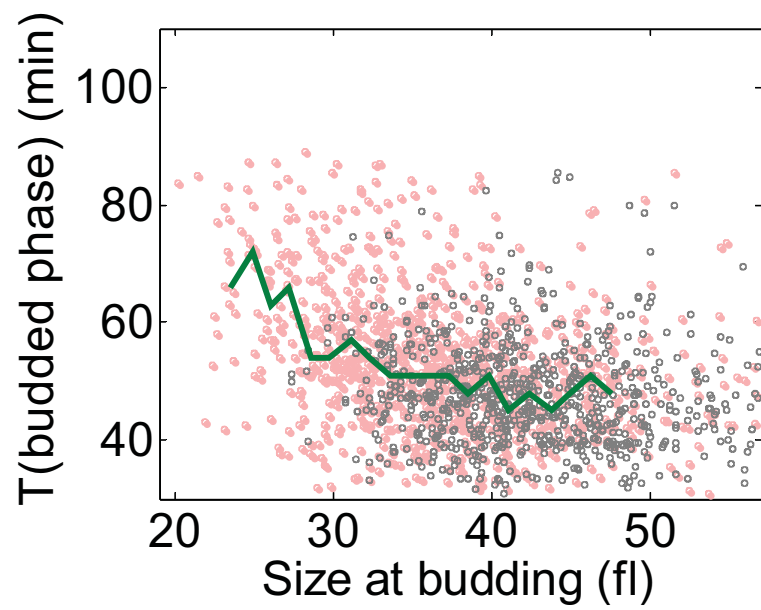

Supplement: Supplementary file 8 — Supplementary Figure S8 [file msb0010-0761-sd8.pdf]
