# Supplementary material for: Systematic identification of cell size regulators in budding yeast
Source: Mol Syst Biol. 2014 Nov 19;10(11):761. doi: 10.15252/msb.20145345 (PMC4299602; doi:10.15252/msb.20145345)
Supplement: Supplementary file 4 — Supplementary Figure S4 [file msb0010-0761-sd4.pdf]

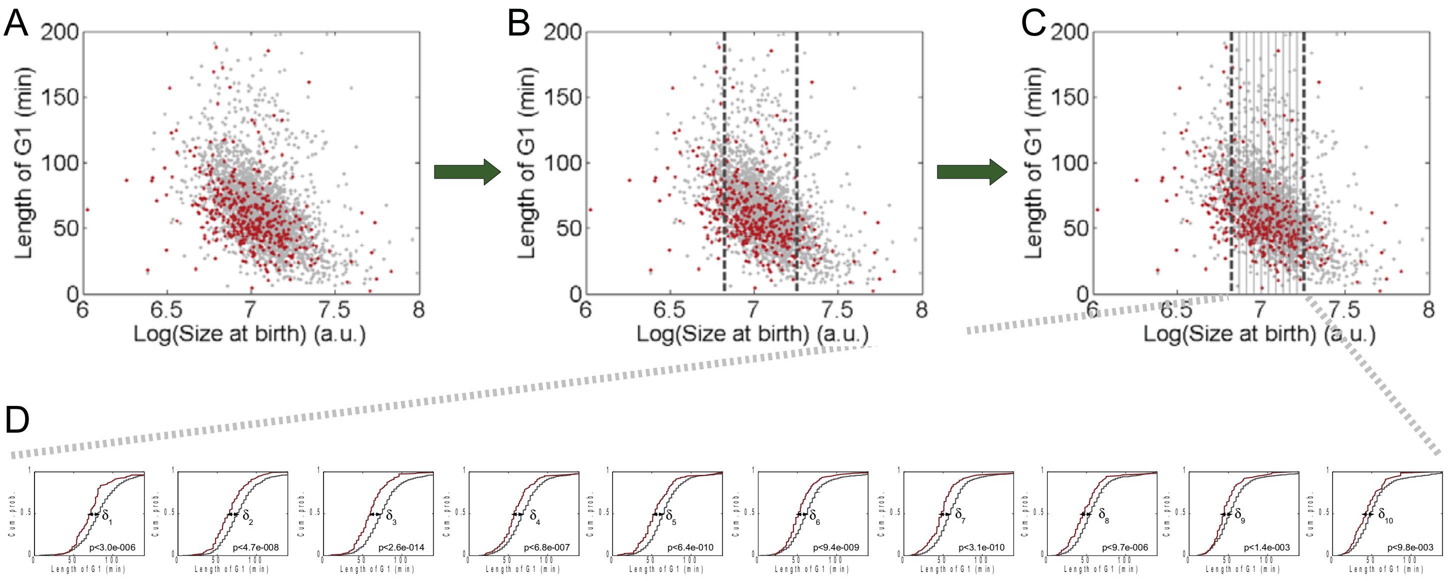

E

Calculate
average relative length of G1

δ=Average(δ_i)

Unite p-values

$$\chi^2_{2k} \sim -2 \sum_{i=1}^{10} \ln(p_i), k = n_{bins}$$
