# Supplementary material for: Systematic identification of cell size regulators in budding yeast
Source: Mol Syst Biol. 2014 Nov 19;10(11):761. doi: 10.15252/msb.20145345 (PMC4299602; doi:10.15252/msb.20145345)
Supplement: Supplementary file 9 — Supplementary Information [file msb0010-0761-sd9.docx]

# Systematic identification of cell size regulators in budding yeast using high throughput time lapse microscopy

Ilya Soifer^1,2^ and Naama Barkai^1^*

^1^Department of Molecular Genetics, Weizmann Institute of Science, Rehovot, 76100, Israel

^2^Present address: NRGENE LTD, 3 Golda Meir, Ness Ziona, Israel

*Corresponding author

# Supplementary Text

## Strain construction

Strain Y8205 was a gift from C. Boone. IS003 (wild type strains for the microscopic screen) was constructed from Y8205 in three steps. First, we transformed Y8205 with a PCR product containing eGFP::kanMX amplified from a plasmid pFA6a-GFP(S65T)-kanMX6 with 40 bp homology to the C-terminus of CDC10 on both sides of the product. Next, we switched kanMX with natMX using a switcher plasmid p4339 (Tong and Boone, 2006). Finally, we transformed the resulting strain with a PCR product containing mCherry::hphMX amplified from a plasmid pBS35 (obtained from the Yeast Resource Center, University of Washington) with 40 bp homology to the C-terminus of ACS2 on both sides of the product.

## High throughput time-lapse microscopy

Olympus IX71 microscope was automated using motorized XY stage (Prior Inc.), fast laser autofocus attachment (Paran *et al*, 2007), excitation and emission filter wheels (Prior Inc.) and shutters (Uniblitz Inc.). The EMCCD camera was Andor iXon with pixel size of 16 $\mu m$ and 512x512 EMCCD chip cooled to -68C. eGFP and mCherry were detected using EXFO X-Cite 120 light source at 12.5% intensity using Chroma 89021 mCherry/GFP ET filter set. Exposure time for the detection of eGFP and mCherry was 100 msec. The cells were observed using 60x0.9 NA UPLFLN/APO objective. The microscope was controlled by custom written software running on Red Hat Linux. The fast auto focus and filter switching times allowed simultaneous imaging of 60 fields of view with time resolution of 3 minutes.

Preparation of cells for time lapse imaging was performed essentially as previously described (Bean *et al*, 2006) but modified for 96-well plate template, that allowed looking at many cultures in parallel. Log stage cultures were seeded at OD 1 on a slab of 2% low melt agarose containing SC and imaged between the agar pad and the cover glass. Bright field images were taken 1 micron below the focal plane to facilitate image analysis. Cells in our setup grow exponentially with the wild type doubling time of around 100 min (Figure S2A).

Our time-lapse setup allows unperturbed exponential growth in a single plane throughout the most of the experiment. Similar to other studies (Bean *et al*, 2006), we note, however, very few cells with extremely long budded period that may be a result of the photo damage due to illumination. These cases, however, do not have a significant effect on our conclusions.

## Image analysis

Acquisition of thousands of cell cycles in a single experiment required fully automated image analysis software. To this end, we developed software that tracks cells throughout the movie series and identifies events of the cell cycle based on the location of the nuclear and the appearance and disappearance of the bud neck marker. The image analysis process consists of three steps as described below.

### Segmentation of the last brightfield image

We process the movie from the last image (Figure S2B) to the first (Cohen *et al*, 2008). As the initial step the last bright field image is segmented. First, morphological reconstruction using Matlab *imreconstruct* function is performed. This step converts the cell interior to become uniformly bright and the cell circumference into uniformly black (Figure S2B). Next, 2D order statistics filter (using the *ordfilt* function) is applied to the output of the previous step imposing the lowest value on each pixel block. This expands the dark boundaries leaving the centers of the cell bright. Finally, the algorithm searches for the local maxima of the resulting image using $imextendedmax$ function to find the cell centers.

The centers identified in the last stage together with the nuclear centers act as seeds for the tracking. This procedure typically identifies >95% of cells in the last image except for the cells that are located in the middle of a colony that may grow out of focus.

### Tracking of the cells along the movie

Cells along the movie are tracked iteratively by using the centers of the cells that were found in the previous step as the seeds for finding the contours of the cells in the current stage. We correct for the imprecision in the XY return position (typically less than 5 pixel distance) by cross-correlating between the consecutive images. The cell contours are found by a dynamical programming algorithm (Kvarnstrom *et al*, 2008) that finds dark polygonal closed line around bright cells. The algorithm finds the best 64-gon that surrounds the cell. The cell volume is estimated by fitting an ellipse to the polygon and then calculating the volume assuming that the cell is a prolate ellipsoid (Figure 2B).

### Identifying the events of the cell cycle

Following the initial tracking of cell along the movie, events of the cell cycle are identified. Fluorescent markers enable identification of three events in the cell cycle: the cytokinesis, marked by the disappearance of the bud neck ring, the end of the G1 phase, marked by the appearance of the bud neck ring, and the metaphase to anaphase transition, marked by separation of the nucleus into two.

To identify the disappearance and the appearance of the bud neck ring automatically, we identify bud neck rings on each figure and assign them to the cell objects. Since in crowded colonies the cell to which the bud neck belongs cannot be identified unambiguously, each identified bud neck is tracked using u-track package (Jaqaman *et al*, 2008) and the cases where the bud neck can be assigned to a cell with certainty are looked for. The background fluorescence is estimated from the regions of the field of view without cells. The intensity of the bud neck is estimated using a sum of 9 brightest pixels of the connected component around the center of the bud neck. The disappearance of the bud neck is determined by a decrease of a bud neck intensity by 30% of its value (after subtracting the background and the camera dark reads) around the time of nuclear separation. Existence of the nuclear marker facilitates the recognition of the bud neck disappearance as its intensity fluctuates a lot during the cell cycle. The typical precision of this procedure is approximately 1-2 figures (3-6 minutes) which is similar to manual determination of the bud neck disappearance. Bud neck appearance is typically easy to determine.

## Selection of strains for the microscopic screen

For the time-lapse microscopy screen we defined six different (partially overlapping) lists (see below, and Dataset E2), introduced fluorescent reporters by SGA methodology and measured cell cycle distribution of the resulting strains. If the cell cycle distribution was significantly different from the original strains (e.g. only diploid colonies recovered after the SGA, or no progeny recovered), we repeated the SGA.

### Negative regulators based on flow cytometry size measurement

We plotted median forward scatter versus percentage of cells in G1 for each mutant in the main screen. This produced a negative correlation as shown on Figure 1F. We neutralized this negative correlation and selected all strains that had forward scatter below expected from the negative correlation between the G1 percentage and forward scatter. Then we selected these strains and measured their cell size and cell cycle distribution. In this repeat every plate contained 4 controls of the wild type strain. Only strains that had the median forward scatter lower than the lowest repeat of the wild type strain were taken for the microscopic screen. Candidate strains were ordered by increasing G1 percentage and for the microscopic screen we took all strains with percentage of G1 phase below that of the wild type (150 strains). 12 strains did not maintain their phenotype after SGA.

### Positive regulators based on flow cytometry size measurement

We plotted median forward scatter versus percentage of cells in G1 for each mutant in the main screen. This produced a negative correlation as shown on Figure 1F. We neutralized this negative correlation and selected all strains that had forward scatter above expected from the negative correlation between the G1 percentage and forward scatter. Then we selected these strains and measured their cell size and cell cycle distribution. In this repeat every plate contained 4 controls of the wild type strain. Only strains that had the median forward scatter above the highest repeat of the wild type strain were taken for the microscopic screen. Candidate strains were ordered by decreasing G1 percentage and for the microscopic screen we took all strains with percentage of G1 phase at least 15% above that of the wild type (194 strains). 20 strains failed to maintain their size after the SGA

### Negative regulators based on Coulter counter size measurement

We plotted median cell size from (Jorgensen *et al*, 2002) versus percentage of cells in G1 for each mutant in the main screen. This also produced a negative correlation. We neutralized this negative correlation and selected all strains that had cell size below the expected from the negative correlation between the G1 percentage and median cell size. Candidate strains were ordered by increasing G1 percentage and for the microscopic screen we took all strains with percentage of G1 phase below that of the wild type (136 strains). 17 strains did not maintain their phenotype after SGA.

### Positive regulators based on Coulter counter size measurement

We plotted median cell size from (Jorgensen *et al*, 2002) versus percentage of cells in G1 for each mutant in the main screen. This also produced a negative correlation. We neutralized this negative correlation and selected all strains that had cell size above the expected from the negative correlation between the G1 percentage and median cell size. Strains that had the median forward scatter lower than the lowest repeat of the wild type strain were taken for the microscopic screen. Candidate strains were ordered by increasing G1 percentage and for the microscopic screen we took all strains with percentage of G1 phase below that of the wild type (115 strains). 30 strains did not maintain their phenotype after SGA.

### Known regulators of cell size and cell cycle

All *whi* strains from (Jorgensen *et al*, 2002),*whi* and *lge* strains from(Zhang *et al*, 2002) and all strains deleted of genes that have GO annotation “regulation of cell size". (103 strains, 19 failed SGA)

### Genes implicated in ribosomal biogenesis

Deletions of all non-essential genes that were annotated with GO term 'ribosomal biogenesis' or its descendants as of May 7, 2009 (117 strains, 7 did not undergo SGA).

## Theoretical analysis of the weak size control model

### Assumptions and notation

In the following sections, we will use the following notation:

| $V^{i}$ | Volume of a cell at birth at generation $i$ |
| --- | --- |
| $V_{t}^{i}$ | Volume of a cell at division at the end of the $i$th cell cycle (i.e. before generation $i+1$) |
| $T^{i}$ | Duration of the $i$th cell cycle |
| $v^{i}$ | $\log V^{i}$ |
| $\Delta v_{g}^{i}$ | Volume gain due to growth at generation $i$, $\Delta v_{g}^{i}\equiv\log\frac{V_{t}^{i}}{V^{i}}$ |
| $\Delta v_{l}^{i}$ | Volume loss due to division at generation $i$, $\Delta v_{l}^{i}\equiv\log\frac{V^{i+1}}{V_{t}^{i}}$ |
| $\varepsilon^{i}$ | Net volume gain/loss at generation $i$, $\varepsilon^{i}\equiv\Delta v_{g}^{i}-\Delta v_{l}^{i}$ |
| $\mu_{i}$ | Volume growth rate in log scale at generation $i$ |

We also assume that cell grows exponentially with the rate $\mu$ throughout the complete cell cycle, i.e.

$$V(t)=V_{0}e^{\mu t}$$

where $V_{0}$ is the initial volume. At the division, the cell divides with mean constant ratio $k$. That is, if we consider one of the two newborn cells after the $i$th cell cycle, its volume will be given by

|  | $V^{i+1}=kV^{i}e^{\mu_{i}T^{i}}.$ | (1) |
| --- | --- | --- |

Alternatively, we can write equation (1) in log scale in the form

$$v^{i+1}=v^{i}+\mu_{i}T^{i}-\log\frac{1}{k}\equiv v^{i}+\Delta v_{g}^{i}-\Delta v_{l}^{i}\equiv v^{i}+\varepsilon^{i}.$$

The dynamics of this equation depends on the mean value of $\varepsilon^{i}$. If $\varepsilon^{i}>0$ on average, the volume of the cell population will grow to infinity. If it is lower than zero, the mean volume will decrease, and if it equals zero, the mean volume remains constant. In what follows, we will show that the volumes distribution of a cell population that divides according to the dynamics that we describe in the main text, will eventually converge to a steady state distribution (i.e. $E(\varepsilon^{i})\to0$ for a sufficiently large $i$) (section ‎0).

Importantly, even if the population tunes $\varepsilon^{i}$ to have a zero mean, the volume distribution will still expand between generations with a diffusion-like dynamics to a variability in $\Delta v_{g}$ and $\Delta v_{l}$.

### Integral feedback analogy and the proof of convergence to a stable steady state

Let $v^{i}$ be the size of the cell born at generation $i$ (since cells are dividing into two we will consider the random one of the two cells or, in case of budding yeast, the daughter cell of generation $i$). Suppose also that the gain of the volume

|  | $\Delta v_{g}^{i}=F(v^{i},\mu)$ | (2) |
| --- | --- | --- |

for some monotonically decreasing smooth $F$ so that $-1<\frac{\partial F}{\partial x}(x,y)<c<0$, that the (exponential) volume growth rate $\mu$ is constant and that at division the volume loss $\Delta v_{l}^{i}$ is constant (Figure S6a). Then let

|  | $v^{i+1}-v^{i}=\varepsilon^{i}=\Delta v_{g}^{i}-\Delta v_{l}^{i}$ | (3) |
| --- | --- | --- |

and since $v^{i+1}-v^{i}=\varepsilon^{i}$ we have $v^{i+1}=\sum_{j=0}^{i} \varepsilon^{j}+v^{0}.$ We will show that $\varepsilon^{i}\to0$. Passing to continuous time $t$ we can rewrite

|  | $v(t)=\int_{0}^{t} \varepsilon(\tau)d\tau+v^{0}.$ | (4) |
| --- | --- | --- |

Substituting (2) and (4) into (3) we have

|  | $\varepsilon(t)=F(\int_{0}^{\tau} \varepsilon(\tau)d\tau+v^{0},\mu)-\Delta v_{l}.$ | (5) |
| --- | --- | --- |

The system may be seen as the implementation of the integral feedback controller aimed at setting $\varepsilon(t)=0$. In this representation, $F$ is a controller whose gain is set by an integral of the previous errors, which is the definition of integral feedback controller. The fact that the error always converges to zero is a basic result in control theory. The volume $v=\int\varepsilon(\tau)d\tau+v^{0}$ at which $0=\varepsilon(t)=F(v,\mu)-\Delta v_{l}$ is $v^{ref}(\mu)$. We will show the proof of this fact in the particular case.

Differentiating w.r.t $t$ we have

|  | $\varepsilon^{'}(t)=F^{'}(\int_{0}^{t} \varepsilon(\tau)d\tau+v^{0},\mu)\varepsilon(t)$ | (6) |
| --- | --- | --- |

and

$$\frac{\varepsilon^{'}}{\varepsilon}=F^{'}(\int_{0}^{t} \varepsilon(\tau)d\tau+v^{0},\mu)$$

(we are allowed to divide by $\varepsilon(t)$ since $\varepsilon(t)=0$ is a steady state point and means that the volume is constant). By the property of $F^{'}$ we have

$$-1<\frac{\varepsilon^{'}}{\varepsilon}<c<0$$

therefore

$$\varepsilon^{-t}<\varepsilon(t)<\varepsilon^{ct}, c<0$$

which means that the error is bounded by two functions that converge to zero and thus converges to zero as required.

We note also that close to steady state where $v^{i}\approx v^{ref}$, $v^{i+1}$ can be written as

|  | $v^{i+1}\approx kv^{i}+c$ | (7) |
| --- | --- | --- |

where $k=1-\frac{\partial F}{\partial v}(v^{ref},\mu)$.

**Supplementary Figure Legends**

**Figure S1. Small strains and strains that have extended G1 are deficient in translation, large strains and strains with extended G2 are deficient in multiple functions that are centered on cell cycle progression in S and G2 phases.**

Enrichment of GO functional category in strains classified as

(A) extended G1 phase,

(B) extended G2 phase,

(C) increased size,

(D) decreased size and

(E) strains with a complex cell cycle profile.

Numbers indicate the number of genes of this functional category that belong to the class, total number of genes in the category and the fold enrichment respectively.

(F) Spearman correlations between the median forward scatters/microscopic volume estimates/electronic volume estimates from this screen and previous screens (Jorgensen *et al*, 2002; Ohya *et al*, 2005; Hoose *et al*, 2012) and between percentages of G1 cells or percentage of unbudded cells measured in this screen and previous screens.

**Figure S2. Characterization of image acquisition and analysis system**

1. Growth of cells in time-lapse movie. The doubling time for the fluorescent movie was calculated by counting nuclei. The doubling time for bright field movie was calculated by counting cell bodies at every time point every 15 minutes. Note that the growth rate with and without illumination with mercury lamp is the same.
2. Initial segmentation and tracking. Top row left to right: the initial image, the image after the morphological reconstruction, the image after order filter. See Supplementary Text for details.

**Figure S3. Properties of size control on different carbons sources.**

1. Volume growth rate in G1 estimated assuming exponential growth plotted versus the initial cell size.
2. Volume growth rate in G1 estimated assuming linear growth plotted versus the initial cell size.
3. Cells that have a higher volume growth rate bud at a larger size. Correlation between the final size and the growth rate for cells born at a similar size.
4. Distribution of sizes at budding for cells born at similar size (log(V_birth_) between 10.8 and 11.2 a.u.) on different media. Note different sizes at budding consistent with different specific growth rates.
5. Distributions of lengths of G1 for cells born at similar size (log(V_birth_) between 10.8 and 11.2 a.u.) on different media. Note similar lengths of G1.
6. Distribution of growth rates of repeats of the wild-type and the mutant strains on glucose.

**Figure S4. Step by step description of quantitative metric of size control**

1. Plot the length of G1 versus the size at birth for the mutant and the wild type strains,
2. Find the interval that contains 80% of the mutant and the wild-type cells,
3. Split the interval into 10 bins,
4. For every bin calculate the p-value of the hypothesis that the medians of the G1 lengths of the mutant and the wild-type are the same using Wilcoxon rank sum test,
5. Calculate the average difference of medians and the unified p-value for the hypothesis that the medians are the same over all bins using Fischer's method,

**Figure S5. Examples of indirect regulators of cell size.** Each subplot contains comparison of the dependency of length of G1 and ΔV in G1 on birth size and histogram of birth sizes and budding sizes of large strains (A) *akr1* and (B) *dbp3* that bud at a large size only because of large size at birth and of small strains (C) *swe1* and (D) *tom1* that are budding at small size because of small birth size.

**Figure S6. Additional size control plots for negative regulators of G1/S transition.** Analysis as in Fig 5.

**Figure S7. Additional plots comparing deletions of large and small ribosomal subunits.**

1. Box plot comparing average population size of wild-type strain (repeats) and deletions of parts of small and large subunits in the microscopy screen. All differences are significant with p-value < 0.001.
2. Box plot comparing average population size of deletions of parts of small and large subunits (normalized to the median population size of wild-type) in the screen by (Jorgensen *et al*, 2002). Deletions of parts of the large subunit were smaller with p-value < 0.001.
3. Box plot comparing average population size of deletions of parts of small and large subunits in the screen by (Ohya *et al*, 2005). All differences are significant with p-value < 0.001.
4. Comparison between measurements of relative ΔV in G1, relative G1, size at birth and size at budding between the repeats of all ribosomal genes, deletions in small subunits and deletions of large subunits of the ribosome. Correlation coefficients are 0.55-0.75.
5. Duration of mother cells cell cycle (with very short G1) relatively to the wild-type in all deletions of the small subunit of the ribosome (green) and deletions of the large subunit of the ribosome (blue).

**Figure S8. Backup mode of size control in small mother cells.**

Shown are the durations of the budded phase in mother cells in the wild-type (gray) and cells of small mutants (pink). Data for the wild-type and small mutants was binned into equally sized bins and plotted (red and green lines respectively).

**Supplementary Datasets**

**Dataset S1. Results of cell cycle measurements of the strains in the basic screen** (All genes tab), and repeats of strains suspected to be large, small, extended G1, extended G2, extended S and also strains that were re-measured due to a special interest (cell cycle involvement etc.). The cell cycle data was normalized so that every mutant plate had the same median and standard deviation and relative amount of cells in each stage normalized by an average percentage is reported. (See Materials and methods). Dataset also shows results from other screens for comparison.

**Dataset S2. List of strains measured, reason that they were selected and result of measurement (success or number of failures in SGA).**

**Dataset S3: Summary of the cell cycle dynamics measured in the screen.** Strains falling into the phenotypic categories as in Fig 4 are colored as follows: green – deletion of negative regulator, blue – deletion of a gene that has indirect effect on size due to slow growth, red – deletion of the positive regulator, violet – strain that is large due to extension of different cell cycle phases.

**Supplementary Tables**

| Condition | Study | r(v_b_, ΔV) | % variance in Δ in G1 explained | r(vb, vs) | Slope (vb, vs) |
| --- | --- | --- | --- | --- | --- |
| Haploid, 2% glucose | This work | -0.63 | 40 | 0.72 | 0.7 |
|  | Di Talia | -0.54 | 32 | 0.72 | 0.62 |
|  | Ferrezuello | -0.34 | 12 | N/A | 0.61 |
| Diploid, 2% glucose | This work | -0.6 | 36 | 0.78 | 0.72 |
|  | Di Talia | -0.62 | 39 | 0.72 | 0.68 |
|  | Ferrezuello | N/A | N/A | N/A | N/A |

**Table S1.** Comparison of the effect of birth size on ΔV in G1 (v_s_-v­_b_) where v_s_ and v_b_ are volumes at budding and birth respectively in this work and (Di Talia *et al*, 2007; Ferrezuelo *et al*, 2012). The effect is measured as the Pearson correlation coefficient (square root of the fraction of variance in ΔV in G1 explained). We also compare the effect of birth size on size at budding (or at Start where applicable) in terms of slope of the linear fit and the Pearson correlation.

|  | Glucose | | 0.05% glucose | | Raffinose | | Galactose | |
| --- | --- | --- | --- | --- | --- | --- | --- | --- |
| H_0_ | $\boldsymbol{v}_{\boldsymbol{s}}^{\boldsymbol{i}}\mathbf{=}\boldsymbol{v}_{\boldsymbol{s}}^{\boldsymbol{j}}$ | $\boldsymbol{T}_{\boldsymbol{G}\mathbf{1}}^{\boldsymbol{i}}\mathbf{=}\boldsymbol{T}_{\boldsymbol{G}\mathbf{1}}^{\boldsymbol{j}}$ | $\boldsymbol{v}_{\boldsymbol{s}}^{\boldsymbol{i}}\mathbf{=}\boldsymbol{v}_{\boldsymbol{s}}^{\boldsymbol{j}}$ | $\boldsymbol{T}_{\boldsymbol{G}\mathbf{1}}^{\boldsymbol{i}}\mathbf{=}\boldsymbol{T}_{\boldsymbol{G}\mathbf{1}}^{\boldsymbol{j}}$ | $\boldsymbol{v}_{\boldsymbol{s}}^{\boldsymbol{i}}\mathbf{=}\boldsymbol{v}_{\boldsymbol{s}}^{\boldsymbol{j}}$ | $\boldsymbol{T}_{\boldsymbol{G}\mathbf{1}}^{\boldsymbol{i}}\mathbf{=}\boldsymbol{T}_{\boldsymbol{G}\mathbf{1}}^{\boldsymbol{j}}$ | $\boldsymbol{v}_{\boldsymbol{s}}^{\boldsymbol{i}}\mathbf{=}\boldsymbol{v}_{\boldsymbol{s}}^{\boldsymbol{j}}$ | $\boldsymbol{T}_{\boldsymbol{G}\mathbf{1}}^{\boldsymbol{i}}\mathbf{=}\boldsymbol{T}_{\boldsymbol{G}\mathbf{1}}^{\boldsymbol{j}}$ |
| Glucose | 1 | 1 | <10^-8^ | 0.61 | <10^-4^ | 0.003 | <10^-9^ | 0.14 |
| 0.05% glucose |  |  | 1 | 1 | <10^-6^ | 0.001 | <10^-8^ | 0.22 |
| Raffinose |  |  |  |  | 1 | 1 | <10^-10^ | 0.12 |
| Galactose |  |  |  |  |  |  | 1 | 1 |

**Table S2.** p-value for the two null hypotheses: (1) if (birth-size normalized) G1 duration $\left( T_{G1} \right)$ is equal between two conditions (i and j) and (2) if the (birth size normalized) volume increase during G1 $v_{s}-v_{b}$ is equal between the two conditions. The test was performed as described in Materials and methods. Note that the differences in the durations of G1 for cells born at the same size in different carbon sources are insignificant (or almost insignificant), while differences in their volume increases in G1 are highly significant.

| Strain | Size in (Jorgensen et al, 2002) | Size at budding in this screen (fl) | Average population size (fl) |
| --- | --- | --- | --- |
| sfp1 | 25 | 23.8 | 47.2 |
| sch9 | 28 | N/A | N/A |
| prs3 | 30 | N/A | N/A |
| whi3 | 31 | 27.0 | 51.1 |
| whi5 | 32 | 23.8 | 47.2 |
| rpa49 | 33 | N/A | N/A |
| ynl227c/jjj1 | 34 | 29.3 | 53.3 |
| ynl226w | 34 | 28.7 | 52.4 |
| pho5 | 34 | 29.8 | 52.6 |
| hxk2 | 34 | 29.5 | 53.2 |
| yhr034c | 35 | 30.9 | 54.6 |
| sky1 | 35 | 30.4 | 60.2 |
| kap122 | 35 | 27.7 | 53.7 |
| ssf1 | 36 | 29.9 | 54.3 |
| rpa14 | 36 | 31.4 | 56.5 |
| ygr111w | 36 | 36.9 | 68.6 |
| ycr061W | 36 | 32.5 | 62.7 |
| ptk2 | 36 | 31.0 | 60.7 |
| tom1 | 37 | 30.4 | 55.9 |
| gpa2 | 37 | 28.7 | 54.3 |
| kel1 | 37 | 30.4 | 55.5 |
| swe1 | 38 | 30.7 | 57.2 |
| ygr064w | 39 | N/A | N/A |
| cdh1 | 40 | 29.5 | 58.5 |
| whi6 | 43 | 34.6 | 63.6 |
| WT | 42 | 33.1 | 61.2 |

**Table S3.** Average and budding sizes of previously identified *whi* strains

| Test null hypothesis | Statistic | Result | p-value |
| --- | --- | --- | --- |
| Size at budding same between RPL and RPS | t-test | Reject H_0_ | 1.6·10^-5^ |
| Size at budding same in RPL and in wt | t-test | Reject H_0_ | 10^-5^ |
| Size at budding same in RPS and in wt | t-test | Reject H_0_ | 10^-11^ |
| Normalized G1 same between RPL and RPS | t-test | Reject H_0_ | 0.007 |
| Normalized ΔV in G1 same between RPL and RPS | t-test | Reject H_0_ | 2·10^-4^ |

**Table S4.** Statistical tests comparing between deletions in the large and small subunits of ribosome.

# Supplementary References

Bean JM, Siggia ED & Cross FR (2006) Coherence and timing of cell cycle start examined at single-cell resolution. *Mol. Cell* **21:** 3–14 Available at: http://dx.doi.org/10.1016/j.molcel.2005.10.035 [Accessed August 3, 2011]

Cohen AA, Geva-Zatorsky N, Eden E, Frenkel-Morgenstern M, Issaeva I, Sigal A, Milo R, Cohen-Saidon C, Liron Y, Kam Z, Cohen L, Danon T, Perzov N & Alon U (2008) Dynamic proteomics of individual cancer cells in response to a drug. *Science (80-. ).* **322:** 1511–1516 Available at: http://www.ncbi.nlm.nih.gov/entrez/query.fcgi?cmd=Retrieve&db=PubMed&dopt=Citation&list_uids=19023046

Ferrezuelo F, Colomina N, Palmisano A, Garí E, Gallego C, Aldea M & Csikász-Nagy A (2012) The critical size is set at a single-cell level by growth rate to attain homeostasis and adaptation. *Nat. Commun.* **3:** 1012 Available at: http://www.nature.com/doifinder/10.1038/ncomms2015 [Accessed August 21, 2012]

Jaqaman K, Loerke D, Mettlen M, Kuwata H, Grinstein S, Schmid SL & Danuser G (2008) Robust single-particle tracking in live-cell time-lapse sequences. *Nat. Methods* **5:** 695–702 Available at: http://www.ncbi.nlm.nih.gov/entrez/query.fcgi?cmd=Retrieve&db=PubMed&dopt=Citation&list_uids=18641657

Jorgensen P, Nishikawa JL, Breitkreutz B-J & Tyers M (2002) Systematic identification of pathways that couple cell growth and division in yeast. *Science (80-. ).* **297:** 395–400 Available at: http://www.ncbi.nlm.nih.gov/pubmed/12089449 [Accessed July 10, 2011]

Kvarnstrom M, Logg K, Diez A, Bodvard K & Kall M (2008) Image analysis algorithms for cell contour recognition in budding yeast. *Opt. Express* **16:** 12943–12957 Available at: http://www.ncbi.nlm.nih.gov/entrez/query.fcgi?cmd=Retrieve&db=PubMed&dopt=Citation&list_uids=18711533

Paran Y, Ilan M, Kashman Y, Goldstein S, Liron Y, Geiger B & Kam Z (2007) High-throughput screening of cellular features using high-resolution light-microscopy; application for profiling drug effects on cell adhesion. *J. Struct. Biol.* **158:** 233–243

Di Talia S, Skotheim JM, Bean JM, Siggia ED & Cross FR (2007) The effects of molecular noise and size control on variability in the budding yeast cell cycle. *Nature* **448:** 947–51 Available at: http://www.ncbi.nlm.nih.gov/pubmed/17713537 [Accessed July 25, 2011]

Di Talia S, Wang H, Skotheim JM, Rosebrock AP, Futcher B & Cross FR (2009) Daughter-specific transcription factors regulate cell size control in budding yeast. *PLoS Biol.* **7:** e1000221 Available at: http://www.pubmedcentral.nih.gov/articlerender.fcgi?artid=2756959&tool=pmcentrez&rendertype=abstract [Accessed July 24, 2011]

Zhang J, Schneider C, Ottmers L, Rodriguez R, Day A, Markwardt J & Schneider BL (2002) Genomic scale mutant hunt identifies cell size homeostasis genes in S. cerevisiae. *Curr. Biol.* **12:** 1992–2001 Available at: http://www.sciencedirect.com/science/article/B6VRT-47F1PMR-J/2/acd6729df87f223b80df1033401b6bb7
